# Clinical Features and Risk Factors of Severe Pneumonia in Children With Acute Lymphoblastic Leukemia

**DOI:** 10.3389/fped.2022.813638

**Published:** 2022-05-06

**Authors:** Chun-yan Liu, Cheng Li

**Affiliations:** ^1^Department of Pediatrics, The Affiliated Hospital of Southwest Medical University, Luzhou, China; ^2^Sichuan Clinical Research Center for Birth Defects, Luzhou, China

**Keywords:** children, acute lymphoblastic leukemia, pneumonia, risk factors, prediction model

## Abstract

**Objective:**

This study aims to analyze the clinical characteristics of pediatric acute lymphoblastic leukemia (ALL) complicated by pneumonia and the risk factors of severe cases to preliminarily construct a prediction model for ALL complicated by severe pneumonia.

**Methods:**

A retrospective analysis was carried out on the clinical data of children diagnosed with ALL complicated by pneumonia hospitalized at the Department of Pediatrics of the Affiliated Hospital of Southwest Medical University between January 2013 and December 2020. The risk factors of severe ALL complicated by pneumonia were investigated with logistic regression analysis, and the risk prediction model was constructed.

**Results:**

A total of 116 cases of pediatric ALL complicated by pneumonia were analyzed. There were 71 cases of mild pneumonia and 45 cases of severe pneumonia. The main clinical manifestations were cough in 112 cases and fever in 109. Pathogens were detected in 23 cases. Multiple regression factor analysis indicated that the use of hormones (OR 4.001, 95% CI: 1.505–10.632), neutropenia or agranulocytosis (OR 7.472, 95% CI: 2.710–20.602), hemoglobin (Hb) < 90 g/L (OR 3.270, 95% CI: 1.256~8.516), and C-reactive protein (CRP) >15 mg/L (OR 3.253, 95% CI: 1.209~8.751) were independent risk factors that were associated with severe pneumonia. Logistic regression was used to establish the risk prediction model of ALL with severe pneumonia. The *p*-value was 0.659. The area under the receiver operating characteristic curve was 0.851, and the sensitivity and specificity were 84.4 and 71.8%, respectively.

**Conclusion:**

The development of severe pneumonia may be affected by the use of hormones, neutropenia or agranulocytosis, Hb < 90 g/L, and CRP > 15 mg/L. The prediction model based on the risk factors is effective, which can provide a reference for the clinical evaluation of acute lymphoblastic leukemia with severe pneumonia.

## Introduction

Pediatric acute lymphoblastic leukemia (ALL) is the most common hematological malignant tumor in childhood (1). Disease-free survival has greatly improved in recent years following improvements in diagnostic techniques and therapeutic regimens. However, because of the nature of the disease and the chemotherapy drugs applied, pediatric patients with ALL are highly vulnerable to concurrent infection, hemorrhage, and toxic or side effects of drugs during treatment. Among these, infection is the most common complication and the leading cause of patients' early death (2). Among all infections, pneumonia is the most common complication during chemotherapy in children with acute lymphoblastic disease, with an incidence of 13% to 31% (3). Bakhshi et al. (4) analyzed 222 Indian children with ALL undergoing chemotherapy at the stage of granular deficiency and found that pneumonia was the most common infection, accounting for ~27.3% of all cases.

Effective and timely treatment for children with ALL complicated by pneumonia arising from chemotherapy is crucial to ensure the best outcome from chemotherapy and increase the survival rate of children with leukemia. In addition, severe pneumonia was the leading cause of death for patients who failed chemotherapy (5). Previous studies mainly focused on hematogenous infection instead of pneumonia. Those that reported pneumonia mostly recruited adults or older patients, rarely pediatric patients. Also, no studies have identified the risk factors for severe pneumonia. Therefore, this study retrospectively sought to analyze the clinical data of 116 children with ALL complicated with pneumonia, analyze the risk factors for severe pneumonia, and establish a preliminarily clinical prediction model to guide clinical treatment and reduce the mortality of children with ALL.

## Subjects and Methods

### Subjects

A retrospective analysis was carried out for 116 children with ALL and pneumonia during chemotherapy who were admitted to a pediatric hematologic oncology group of the Affiliated Hospital of Southwest Medical University from January 2013 to December 2020. The inclusion criteria were as follows: (1) hospitalized between January 2013 and December 2020; (2) aged ≤14 years; (3) diagnosed with ALL and pneumonia; (4) complete clinical data; (5) received at least one course of treatment following the CCLG-ALL2008 program at our hospital from January 2013 to December 2020. (6) All cases with pneumonia occurred during chemotherapy, that is, 48 h after admission. The exclusion criteria were as follows: (1) incomplete clinical data; (2) did not receive chemotherapy. The local Ethics Committee approved this study (approval number: KY2021288).

### Diagnostic Criteria

ALL was diagnosed based on the 2014 Recommendations for Diagnosis and Treatment of Pediatric Lymphoblastic Leukemia (6). The diagnostic criteria and grading of pneumonia were in accordance with the diagnosis and treatment standards for community-acquired pneumonia in children in China. Pneumonia refers to lung parenchyma and/or acute infection in the interstitial parts of the lungs, causing different degrees of hypoxia and infection and poisoning symptoms, such as fever, cough, rapid breathing, dyspnea, chest wall inhalation, moist rales, and tubular breathing sounds in the respiratory tract, and signs of abnormal changes in chest X-rays. The diagnostic criteria for mild pneumonia are patients: (1) in generally good condition; (2) show no signs of refusal to eat and dehydration; (3) have no unconsciousness or hypoxemia; (4) have no extrapulmonary complications; (5) chest X-ray and Computed tomography (CT) results do not meet the criteria of severe pneumonia. The definition of severe pneumonia was clinical manifestations and/or auxiliary studies with any of the following: (1) arterial oxygen saturation (SaO_2_) ≤0.92 under room air conditions; (2) respiratory rate (RR) >70 times/min (infants), RR >50 times/min (others) under room air conditions, except for fever, crying, and other factors; (3) dyspnea: inhalation in the chest wall, fanning of the nose; (4) chest scans and other imaging data confirm that multiple lobes or more than two-thirds of the lungs were involved (7).

### Data Collection

The clinical data of children were collected. The review of individual medical charts was performed for the eligible subjects by trained investigators applying a predesigned case report form. The following data were collected: (1) age, gender, urban vs. rural residence, immunophenotyping, whether or not dexamethasone (6~8 mg/m^2^·d) was used, whether or not red blood cell suspension infusion was done, whether or not blood platelet infusion was done, length of stay, and single vs. multiple-bed rooms; (2) clinical manifestations, symptoms, and signs, including cough, fever, shortness of breath, cyanosis, and results of general physical examination; (3) laboratory tests (performed within 24 h of the occurrence of pneumonia): routine blood tests, C-reactive protein (CRP) levels, sputum cultures, blood cultures, combined detections of nine respiratory pathogens, including respiratory syncytial virus, parainfluenza virus, adenovirus, influenza B virus, influenza A virus, mycoplasma pneumoniae, chlamydia pneumoniae, legionella pulmonary, and seven types of common respiratory pathogen-specific IgM antibodies, as well as chest CT. According to the definition of severe pneumonia, patients were divided into the ALL with mild pneumonia and ALL with severe pneumonia groups.

### Statistical Analysis

Statistical analysis was performed using the SPSS 20.0 software. The normally distributed measurement data were expressed as mean ± standard deviation (x ± s). The non-normally distributed measurement data were expressed as median (upper and lower quartile) (M [P_25_, P_75_]), and rank sum tests were used for comparison among groups. Enumeration data were expressed as percentages, and comparisons among groups were performed using the chi-square test and/or Fisher's exact test. Statistically significant items in univariate analysis were included in binary logistic regression analysis. The risk prediction model was constructed based on SPSS logistic regression, and the ability of the prediction model was evaluated. The Hosmer–Lemeshow goodness of fit test was used to evaluate the prediction model ability. The regression equation was fixed. The PRE was calculated by including each influencing factor and its regression coefficient (β) into the SPSS software, and then the receiver operating characteristic (ROC) curve was used to evaluate the prediction efficiency of the risk model. The significant level was α = 0.050, and *P* < 0.050 was considered statistically significant.

## Results

### Demographics

A total of 116 cases of ALL complicated by pneumonia meeting the requirements were included in the study, including 68 males (58.6%) and 48 females (41.4%). The average age of the patients was 5.3 years. The rate of multi-bed rooms and hormone use in the ALL severe pneumonia group was significantly higher than in the ALL mild pneumonia group, and the difference was statistically significant (Table 1).

**Table 1:** Comparison of general data between the ALL with mild pneumonia group and the ALL with severe pneumonia group.

**General data**	**Total (*N* = 116)**	**ALL with mild pneumonia group** **(*N* = 71)**	**ALL with severe pneumonia group** **(*N* = 45)**	**χ^2^/*Z***	***P***
Male	68 (58.6%)	41 (57.8%)	27 (60.0%)	0.058	0.810
Age		4.7 (3.4~7.5)	4.0 (2.7~6.4)	−1.439	0.150
Place of residence				1.407	0.236
Urban areas	57 (49.1%)	38 (53.5%)	19 (42.2%)		
Rural areas	59 (50.9%)	33 (46.5%)	26 (57.8%)		
Bed number				5.344^*^	0.021
Multiple beds room	54 (46.6%)	27 (38.0%)	27 (60.0%)		
Single bed room	62 (53.4%)	44 (62.0%)	18 (40.0%)		
Length of stay				0.753	0.385
Length of stay ≥ 14 days	56 (48.3%)	32 (45.1%)	24 (53.3%)		
Length of stay < 14 days	60 (51.7%)	39 (54.9%)	21 (46.7%)		
Immunophenotyping				0.265	0.607
B-cell type ALL	93 (80.2%)	58 (81.7%)	35 (77.8%)		
T-cell type ALL	23 (19.8%)	13 (18.3%)	10 (22.2%)		
Use of hormones	57 (49.1%)	25 (35.2%)	32 (71.1%)	14.203^*^	0.000
Received red blood cell suspension infusion	36 (31.0%)	18 (25.4%)	18 (40.0%)	2.761	0.097
Received blood platelet transfusion	41 (35.3%)	21 (29.6%)	20 (44.4%)	2.664	0.103

* *P < 0.050*.

### Clinical Manifestations

Among the 116 cases, 109 (94.0%) had a fever, 112 (96.6%) had a cough, 60 (51.7%) had wheezing, and 83 (71.6%) had moist pulmonary crackles. In the 45 patients in the ALL and severe pneumonia group, 41 cases (91.1%) had polypnea, 42 (93.3%) had cyanosis, 29 (64.4%) had dyspnea, 18 (40.0%) had disturbance of consciousness, and 21 (26.7%) had respiratory failure. The clinical manifestations of the two groups are shown in Table 2.

**Table 2:** Comparison of clinical manifestations and auxiliary studies between ALL patients with mild pneumonia and ALL patients with severe pneumonia.

**Clinical manifestations/Auxiliary examination**	**Total** **(*N* = 116)**	**ALL with mild pneumonia group** **(*n* = 71)**	**ALL with severe pneumonia group** **(*n* = 45)**	**χ^2^**	***P***
Fever	109 (94.0%)	65 (91.5%)	44 (97.8%)	1.884	0.170
Cough	112 (96.6%)	68 (95.8%)	44 (97.8%)	0.332	0.565
Wheezing	60 (51.7%)	40 (56.3%)	20 (44.4%)	1.560	0.212
Moist pulmonary Crackles	83 (71.6%)	47 (66.2%)	36 (80.0%)	2.578	0.108
Polypnea	41 (35.3%)	0	41 (91.1%)	–	0.000
Cyanosis	42 (36.2%)	0	42 (93.3%)	–	0.000
Dyspnea	29 (25.0%)	0	29 (64.4%)	–	0.000
Disturbance of consciousness	18 (15.5%)	0	18 (40.0%)	–	0.000
Respiratory failure	12 (10.3%)	0	12 (26.7%)	–	0.000
Neutropenia or agranulocytosis	59 (50.9%)	25 (35.2%)	34 (75.6%)	17.938	0.000
HB <90 g/L	51 (44.0%)	23 (32.4%)	28 (62.2%)	9.947	0.002
PLT <100 × 10^9^/L	60 (51.7%)	35 (49.3%)	25 (55.6%)	0.432	0.511
CRP > 15 mg/L	58 (50.0%)	27 (38.0%)	31 (68.9%)	10.493	0.001
Positive bacterial culture	19 (16.4%)	13 (18.3%)	6 (13.3%)	0.498	0.480
Fungi	1 (0.9%)	0	1 (2.2%)	–	0.388
Parainfluenza virus	2 (1.7%)	2 (2.8%)	0	–	0.521
Mycoplasma	1 (0.9%)	1 (1.4%)	0	–	1.000
**Changes in chest X-ray/CT findings**					
Ground-glass opacity	31 (26.7%)	20 (28.2%)	11 (24.4%)	0.159	0.659
Patchy, blurred opacity	48 (41.4%)	31 (43.7%)	17 (37.8%)	0.393	0.531
Nodular shadows	13 (11.2%)	7 (9.9%)	6 (13.3%)	0.334	0.563
Solid changes	22 (19.0%)	13 (18.3%)	9 (20.0%)	0.051	0.821
Solid changes with pleural effusion	2 (1.7%)	0	2 (4.4%)	–	0.148

* *P < 0.050. The “–” item is Fisher's exact probability method, without statistical value; Neutropenia (agranulocytosis): <1 year. The absolute value of neutrophils <1.000 × 10^9^/L; ≥1 year. The absolute value of neutrophils <1.500 × 10^9^/L. Neutropenia (agranulocytosis): The absolute value of neutrophils <0.500 × 10^9^/L*.

### Auxiliary Studies

Among the 116 cases, 59 (50.9%) had hypogranulosis or granular deficiency, 51 (44.0%) had hemoglobin (Hb) <90 g/L, 60 (51.7%) had blood platelet (PLT) < 100 × 10^9^, and 58 (50.0%) had CRP > 15 mg/L. A total of 23 (19.8%) were positive for pathogens, including 19 (16.4%) for bacteria, one (0.9%) for fungus, two (1.7%) for parainfluenza virus, and one (0.9%) for mycoplasma. All 116 children underwent chest film or CT examinations, and the main changes were in the bilateral lungs. Among them, 31 cases (26.7%) were mainly changed by ground-glass shadows, 48 (41.4%) were mainly patchy blurred shadows, 13 (11.2%) were nodular shadows, 22 (19.0%) were solid changes, and two (1.7%) were solid changes with pleural effusion. The incidences of granulocytopenia, Hb < 90 g/L, and CRP > 15 mg/L in the ALL with severe pneumonia group were significantly higher than those in the mild pneumonia group, and the difference was statistically significant (*P* < 0.050). The other test results showed no significant difference between the mild and severe pneumonia groups (Table 2).

### Results of Logistic Regression Analysis for Children With Leukemia Complicated by Severe Pneumonia

To further analyze the risk factors that may be associated with severe pneumonia during leukemia chemotherapy, single suspicious factors in the two groups, including the number of beds in the ward (multiple beds), use of hormones, and neutropenia or agranulocytosis, Hb < 90 g/L, and CRP > 15 mg/L, were screened and then included in the binary logistic stepwise regression equation. It was concluded through analysis that the use of hormones (χ^2^ = 14.203, *P* = 0.000), neutropenia or agranulocytosis (χ^2^ = 17.938, *P* = 0.000), Hb < 90 g/L (χ^2^ = 9.947, *P* = 0.002), and CRP > 15 mg/L (χ^2^ = 10.493, *P* = 0.001) were independent influencing factors that may cause severe pneumonia (Table 3).

**Table 3:** Multivariate analysis of ALL with severe pneumonia.

**Variables**	***B***	**Standard error**	**Wald**	**Degree of freedom**	***B***	**OR**	**95% confidence interval for OR**
Bed number (Multiple beds room)	0.599	0.494	1.470	1	0.225	1.820	0.691~4.792
Use hormones	1.387	0.499	7.730	1	0.005	4.001	1.505~10.632
Neutropenia or agranulocytosis	2.011	0.517	15.103	1	0.000	7.472	2.710~20.602
HB <90 g/L	1.185	0.488	5.886	1	0.015	3.270	1.256~8.516
CRP > 15 mg/L	1.180	0.505	5.460	1	0.019	3.253	1.209~8.751
Constant	−3.774	0.681	30.733	1	0.000	0.023	

### Risk Prediction Model of ALL With Severe Pneumonia

**Figure 1:**
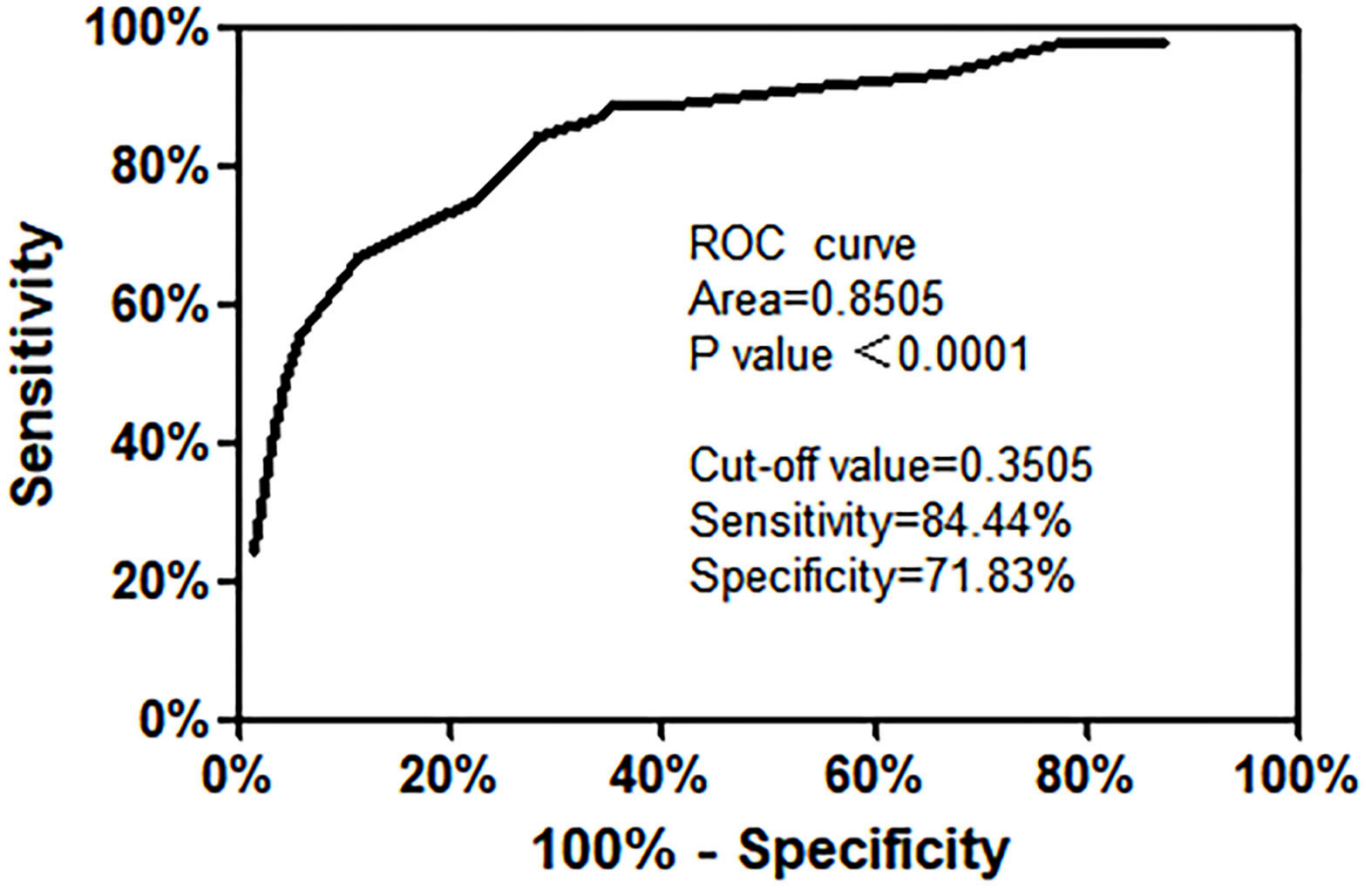
Receiver operating characteristic curve of the risk prediction model for ALL with severe pneumonia.

The risk prediction model was constructed according to the multiple logistic regression analysis results. The significant influencing factors identified in multivariate analysis (shown in Table 3) and regression coefficient B values were included in the Hosmer–Lemeshow goodness of fit test evaluation equation. The model constructed in this study was LogitP = −3.744 + 1.387 × (hormone use) + 2.011 × (neutropenia or agranulocytosis) + 1.185 × (Hb < 90 g/L) + 1.180 × (CRP > 15 mg/L). The Hosmer–Lemeshow goodness of fit test was used to evaluate the combined effect of the equation. The results showed that χ^2^ = 5.009 and *P* = 0.659 > 0.050, suggesting that the difference between the predicted and actual values was not statistically significant, and the prediction model had good calibration ability. The ROC curve was applied to evaluate the effectiveness of the model. The results showed that the area under the curve was 0.851 > 0.75, 95% CI was 0.77–0.925, *P* < 0.050. The best cut-off value of risk prediction was 0.350. When the risk prediction value was higher than 0.350, severe pneumonia was likely to occur in children with ALL, with a sensitivity of 84.4% and a specificity of 71.8% (Figure 1).

### Treatment and Outcomes

All 116 children were treated with antibiotics, among which 82 cases (70.7%) received β-lactams treatment, 24 (20.7%) received carbapenem antibiotics, and 10 (8.6%) received carbapenem antibiotics combined with glycopeptide antibiotics for antifungal therapy. Based on their hemogram, the patients were provided with transfusions of red cells and platelets, and recombinant human granulocyte colony-stimulating factors via intramuscular injection and chemotherapy drugs were stopped. Severe cases were provided with immunoglobulins and other comprehensive treatments. The mild pneumonia group recorded 71 cases (100%) as fully recovered; whereas the severe group registered 42 (93.3%) as fully recovered, one (2.2%) died of pneumorrhagia, and two cases withdrew (4.4%).

## Discussion

Complicated pneumonia is quite common among pediatric patients with ALL who receive chemotherapy, which may prolong their hospital stay and increase the financial burden on their families. In addition, severe pneumonia is often life-threatening. The study carried out a retrospective analysis for 116 pediatric patients with ALL complicated by pneumonia and investigated risk factors that may be associated with severe pneumonia, and a preliminary risk prediction model for severe pneumonia in pediatric ALL was established.

According to the analysis, the most common clinical manifestations of ALL complicated by pneumonia are cough (96.6%), fever (94.0%), moist crackles (71.6%), and wheezing (51.7%). Most severe cases suffered from dyspnea (64.4%), disturbance of consciousness (40.0%), respiratory failure (26.7%), and other complications within 3–5 days, suggesting rapid progression of ALL complicated by severe pneumonia. Therefore, early identification and treatment can help control the progression and improve prognosis. Among the 116 pediatric patients, 19.8% tested positive for pathogen detection. Compared with pneumonia's 50% pathogen detection rate (8), the pathogen detection rate in the current study was lower. This may be partly attributed to the small sample size, collection of some samples that lacked time efficiency, single bottle collection of blood cultures, and non-standard collection of sputum cultures. Pathogens detected in the study were mainly bacteria, such as Streptococcus pneumoniae and Gram-negative bacilli, consistent with previous Hospital-acquired pneumonia (HAP) pathogen studies (9). Lung imaging findings typically showed patchy, blurred opacity, ground-glass opacity, and consolidation shadow. Other changes were rare.

In this study, one person died in the severe pneumonia group, accounting for 2.2%, which was in line with the UKALL2003 infection-related mortality rate (2.4%) (10). Multiple logistic regression analysis indicates that the use of hormones, neutropenia or agranulocytosis, Hb < 90 g/L, and CRP > 15 mg/L were independent influencing factors of severe pneumonia occurrence.

As glucocorticoids can specifically induce apoptosis of malignant lymphocytes (11), they play a key role in the treatment of lymphatic system malignancy (12), especially for ALL, where they are indispensable throughout the course of treatment. The study found that the application of glucocorticoids is also an independent influencing factor that causes severe pneumonia. Previous studies in the literature show that long-term, high-dose use of hormones can lead to severe immunosuppression and make the host more vulnerable to various pathogenic bacteria. As the lung is the most common target organ of infection, mortality surges when the case is complicated by pneumonia (13). Previous evidence has also shown that the use of hormones is significantly associated with lower respiratory tract infections in patients with hematological malignancies (14).

An analysis carried out by the Chinese scholars Lijuan et al. on the case data of 100 patients with pneumonia who received glucocorticoid treatment for a long time in 11 grade A tertiary hospitals showed that patients using long-term hormones recorded a higher severity of pneumonia and in-hospital fatality rates (15).

Neutropenia caused by cytotoxic chemotherapy is a common high-risk factor for concurrent infections in the chemotherapy of hematological malignancies (16). During chemotherapy, granulocytopenia, or granulocytosis have been considered risk factors for pneumonia in children with acute lymphoma (17). In addition, severe neutropenia (<500 cells/μL) was associated with severe pulmonary infection and poor prognosis, highlighting the relevance of these risk factors. It is estimated that nearly 60% of cancer patients with chemotherapy-induced neutropenia develop lung infiltrates on radiographs (17). This is because neutropenia induced by myelosuppression can result in infection, a common post-chemotherapy complication among children with leukemia and a leading cause of death. Long-term, high-dose use of chemotherapy drugs and their metabolites directly disrupts a patient's bone marrow microenvironment and myeloid progenitors, resulting in neutropenia and even agranulocytosis (18). Currently, a wealth of clinical studies on the correlation between agranulocytosis and infectious diseases concluded that the two factors are closely interwoven. The study also shows that neutropenia or agranulocytosis is an independent risk factor that leads to severe pneumonia in children with leukemia. Anemia can damage the immune system of children and aggravate the infection. There is evidence that red blood cells are directly involved in the maintenance of innate and adaptive immune systems and are regulators of T-cell proliferation (19). Therefore, children with anemia are more likely to have severe infections.

CRP is an acute inflammatory protein and a common inflammatory indicator of infection, with unique biological characteristics. On the one hand, CRP activates the classical complement pathway, induces phagocytosis, and promotes cell apoptosis. On the other hand, it promotes chemotaxis of circulating white blood cells and recruitment to inflammatory areas and can delay cell apoptosis (20), so it is significantly increased during infection. Some overseas studies have shown a certain correlation between CRP and the severity of the disease. In children with community-acquired pneumonia, CRP in severe pneumonia is significantly higher than in mild pneumonia (21). Another study showed that CRP was significantly increased in children with acute lymphadenopathy during chemotherapy for severe pneumonia (22). The present study also found that CRP > 15 mg/L was an independent risk factor for severe pneumonia in children with leukemia.

Based on the current findings, we recommend that children with ALL complicated with pneumonia who receive hormones during chemotherapy have their blood routinely monitored. For children with neutropenia or agranulocytosis, and Hb < 90 g/L and CRP > 15 mg/L, early identification of the occurrence of severe pneumonia was important. For CRP > 15 mg/L, higher-intensity intravenous antibiotics should be given with sufficient dosage and frequency to control lung infections early. For patients with Hb < 90 g/L, the degree of anemia must be closely observed, and, if necessary, a transfusion of red blood cell suspension should be given to correct the anemia.

The limitation of this study is that it is a single-center and retrospective study with a relatively small sample size. Therefore, model validation was not performed. The predictive value of the established model for ALL complicated with severe pneumonia still needs further investigation by larger and multi-center studies in the future.

In summary, this study shows that the use of hormones, reduced number of granulocytes, Hb < 90 g/L, and CRP > 15 mg/L may be independent risk factors for severe pneumonia in children with ALL by analyzing their clinical characteristics. The predictive model constructed based on the risk factors can provide a reference for clinical evaluation of ALL with severe pneumonia, improve the early detection of children with high risks, and contribute to early intervention and treatment of severe diseases.

## Data Availability Statement

The original contributions presented in the study are included in the article/supplementary material, further inquiries can be directed to the corresponding author/s.

## Ethics Statement

The studies involving human participants were reviewed and approved by Ethics Committee of The Affiliated Hospital of Southwest Medical University. Written informed consent to participate in this study was provided by the participants' legal guardian/next of kin.

## Author Contributions

C-yL and CL conceived the idea, conceptualized the study, and drafted the manuscript. C-yL collected the data and reviewed the manuscript. CL analyzed the data. Both authors read and approved the final draft.

## Funding

This research was supported by the Scientific Research Project of National Health and Family Planning Commission Medical and Health Technology Development Research Center (W2016EWSC04).

## Conflict of Interest

The authors declare that the research was conducted in the absence of any commercial or financial relationships that could be construed as a potential conflict of interest.

## Publisher's Note

All claims expressed in this article are solely those of the authors and do not necessarily represent those of their affiliated organizations, or those of the publisher, the editors and the reviewers. Any product that may be evaluated in this article, or claim that may be made by its manufacturer, is not guaranteed or endorsed by the publisher.
